# Emergency department-based injury surveillance information system: a conceptual model

**DOI:** 10.1186/s12873-023-00831-9

**Published:** 2023-06-01

**Authors:** Nader Mirani, Haleh Ayatollahi, Davoud Khorasani-Zavareh, Kimia Zeraatkar

**Affiliations:** 1 Department of Health Tourism, Zanjan University of Medical Sciences, Zanjan, Iran; 2 Health Management and Economics Research Center, Health Management Research Institute, Iran University of Medical Sciences, 1996713883 Tehran, Iran; 3 Safety Promotion and Injury Prevention Research Center, Department of Health in Emergencies and Disasters, Shahid Beheshti University of Medical Sciences, Tehran, Iran; 4 Department of Neurobiology, Care Sciences and Society (NVS), Division of Family Medicine and Primary Care, Karolinska Institute, Huddinge, H1 Sweden; 5 Education Development Center, Hormozgan University of Medical Sciences, Bandar Abbas, Iran

**Keywords:** Emergency department, Injury, Injury surveillance information system

## Abstract

**Background:**

Injury data play a pivotal role in monitoring public health issues and Injury Surveillance Information Systems (ISIS) are useful for continuous data collection and analysis purposes. Since emergency department (ED) is usually the first place of referral for the injured people, the aim of this study was to develop a conceptual model for an ED-based ISIS.

**Methods:**

This study was completed in 2020 and the Delphi technique (three rounds) was used to determine the main components of an ED-based ISIS. The participants were selected using the purposive sampling method. A 5-point Likert scale questionnaire was used for data collection and data were analyzed using descriptive statistics.

**Results:**

In the first, second, and third rounds of the Delphi study, 60, 44, and 28 experts participated, respectively. In the first and second rounds, most of the items including the personal data, clinical data, data sources, and system functions were found important. In the third round of the Delphi study, 13 items which did not reach a consensus in the previous rounds were questioned again and five items were removed from the final model.

**Conclusion:**

According to the findings, various data elements and functions could be considered for designing an ED-based ISIS and a number of data sources should be taken into count to be integrated with this system. Although the conceptual model presented in the present study can facilitate designing the actual system, the final system needs to be implemented and used in practice to determine how it can meet users’ requirements.

**Supplementary Information:**

The online version contains supplementary material available at 10.1186/s12873-023-00831-9.

## Introduction

According to the World Health Organization (WHO), injuries (intentional and unintentional) constitute about 8% of all deaths in the world and more than 4.4 million people die due to different types of injuries annually [1]. However, most of injuries are nonfatal, and can be treated in Emergency Departments (EDs) [2]. According to the European Association for Injury Prevention and Safety Promotion (EuroSafe), about 38 million Europeans annually refer to the ED due to injuries and 90% of them suffer from unintentional injuries [3]. These figures suggest that collecting accurate and meaningful injury data are necessary for identifying injury patterns and planning for future interventions [4]. To achieve this, data science and information technology can be applied together to improve data quality which is crucial to monitor injury trends [5].

As injury management process is especially important at the early stages of an accident and most of the injured people are referred to the EDs, the use of Injury Surveillance Information System (ISIS) in this department can help to manage a large volume of clinical and non-clinical data [6]. Such a system can support injury surveillance processes which include collecting, analyzing, and distributing injury data and reports [7–9]. These data can be used by healthcare policymakers to understand the causes of injuries, to determine when and where these injuries occur, and to make the right decisions when developing injury prevention strategies and monitoring injury trends [10]. ISIS is like a mirror that shows a bigger picture of injuries to managers and decision makers [11]. It has been used in different countries for different purposes and covers different geographical areas like a city, a district, or a larger area consisting of several districts or states/provinces [6, 12–20]. This system can help to reduce mortality and disability caused by injuries and support a wide range of services from injury prevention to pre-hospital, in-hospital, and post-discharge care mainly by providing adequate and timely data [21].

As the quality of data and documentation in the EDs are usually affected by the nature of emergency care services which include tasks complexity, high speed healthcare delivery, multiple interruptions, and sometimes dealing with unknown or complex cases [22–26], the use of ED-based ISIS can improve injury data collection in a more standard way [27]. So far, a number of ISISs have been developed based on the data obtained from the emergency departments (EDs). For example, the South Korean ISIS, which was fully implemented in 2005, relies on emergency data and collects, organizes, encodes, and shares data based on the WHO guidelines [12]. In 1996, the first steps were taken by the Egyptian Ministry of Health and Population (MOHP) to launch a national ISIS in Egypt. Since 2010, most Egyptian hospitals are collecting and distributing their injury data within the framework suggested by the Egyptian ISIS [28, 29]. The National Electronic Injury Surveillance System-All Injury Program (NEISS-AIP) was an expansion of NEISS and was established in the USA to collect data, on initial visits, for all types and external causes of nonfatal injuries and poisonings treated in US hospital EDs [30]. The Canadian Hospitals Injury Reporting and Prevention Program (CHIRPP) is an emergency department-based injury and poisoning surveillance system which established the web-based eCHIRPP system in 2011 [31].

An emergency department-based ISIS (ED-based ISIS) can help reducing patient mortality, decreasing costs, speeding up the information management processes, and increasing research in the field of injuries [32, 33]. Improving the timeliness and quality of data which in turn, improves the quality of care is another benefit of using this system [31, 34–36]. Moreover, this system will prevent duplications and wasting resources, and will provide a better context for implementing effective injury control and prevention programs at the regional and national levels [6]. Collecting standard data across all emergency departments can also support comparisons at the regional and national levels [31]. Although several injury surveillance systems have been developed in the world to cover different types of injuries such as sport [16] and child injuries [32], it seems that an ED-based ISIS can be a more useful system to include all types of injuries and provide a more complete dataset for further analysis.

In Iran, the highest incidence of injuries is mainly related to traffic injuries, followed by trauma and falls from heights, respectively [37]. In addition, the lack of a traffic accident surveillance system is one of the most important road safety challenges in Iran [38]. Therefore, in order to close monitoring of different types of injuries including traffic accidents, traumas, etc., designing and implementing an ED-based ISIS can be a solution to manage injury data more effectively. The aim of the current study was to use the Delphi technique to develop a conceptual model for an ED-based ISIS to gain a more complete understanding of the future system.

## Methods

The present study was conducted in 2020. Before conducting the research, ethics approval was sought from the University Ethics committee (IR.IUMS.REC 1394.9221563205). The original study composed of several phases including a literature review [27], interview with the experts, an expert panel and a Delphi study to finalize a conceptual model. After completing the literature review, a number of data elements and functions required for an ISIS were identified. Moreover, the literature review helped to develop the interview guide. In the second phase of the study, 26 experts in the field of injury were interviewed and data elements, data sources, and required functions of the system were identified based on their comments. The experts were emergency medicine specialists, general practitioners, and nurses who worked in six EDs and two trauma research centers. In addition, managers and administrative staff, who worked in the center of accident and emergency services at the medical universities and the Ministry of Health, were invited to take part in the study.

In the third phase of the study, a draft of the conceptual model was developed based on the findings derived from the literature review and interview with the experts. It was presented to an expert panel (*n* = 6) which constituted of emergency medicine specialists (*n* = 3) and managers who worked in the center of accident and emergency services at the Ministry of Health (*n* = 3). These people previously took part in the interviews and were interested to participate in the expert panel, too. They commented on different parts of the conceptual model and it was modified based on their opinions. Then, the Delphi technique (three rounds) was used to validate the conceptual model and its components in a larger sample size. The current study presents the results of the Delphi study.

### Research settings

The research settings included six EDs affiliated to three different medical universities, two trauma research centers, and the center of accident and emergency services at the medical universities and the Ministry of Health. These EDs were the most crowded ones (more than 30,000 patients annually), and every two EDs affiliated to one medical university. Trauma research centers were in two different hospitals. Patient emergency medical records were mainly paper-based and few data elements including patient demographic data, laboratory and radiology tests, and their results were entered into the hospital information systems. The center of accident and emergency services was located in the treatment affaires of three different medical universities and in the Ministry of Health to provide the reports at different levels.

### Participants

The purposive sampling method was used to select the participants of the study who were emergency medicine specialists, general practitioners, and nurses who worked in the EDs and trauma research centers. Other participants were managers and administrative staff who worked in the center of accident and emergency services at medical universities and the Ministry of Health. The selection criteria were having at least three years of work experience in the emergency care services, injury and trauma and in total, 183 people met the criteria to be invited to take part in the study.

### Research instrument

In order to collect data, a five-point Likert scale questionnaire (very important (5), important (4), moderately important (3), slightly important (2), and not important (1)) was developed based on the findings derived from the previous phases of the research [27]. The face and content validity of the questionnaire were assessed by five experts in the fields of health information management and emergency medicine who had not participated in the previous rounds of the study. They were given a draft of the conceptual model and the questionnaire together and asked to indicate whether the questionnaire was developed based on the conceptual model, the arrangement of the items was clear, and the number and wording of the questions/items were appropriate. The final questionnaire consisted of 4 parts as follows: (1) participant’s characteristics (8 questions), (2) personal and clinical data elements (64 questions) 3), data sources including hospital and non-hospital sources (11 questions), and 4) functions of the system (30 questions) (Appendix I). Regarding data elements, the mandatory or optional nature of them was also questioned.

In the second round of the Delphi study, the questionnaire consisted of four parts: (1) participant’s characteristics (8 questions), (2) personal and clinical data elements (34 questions), (3) non-hospital data sources (8 questions), and (4) functions of the system (8 questions). In the third round of Delphi study, the questionnaire consisted of three parts: (1) participant’s characteristics (8 questions), (2) personal data elements (9 questions), and (3) non-hospital data sources (4 questions). In all rounds of the Delphi study, paper-based questionnaires were distributed among the participants and collected by one of the researchers (NM).

### Data analysis

Descriptive statistics (frequency, percentage, mean, standard deviation, and the interquartile range) were used to analyze data for each question. If 75% of the participants or more chose the first two options of the questionnaire for an item (i.e., very important and important), and the mean value was more than 3.75, it could be important and needed to be considered in the final model. Those items for which a total of 50 to 75% of the participants chose the first two options and the mean value was between 2.5 and 3.75, were questioned again in the second round of the Delphi study as the participants had not reached an agreement about their importance, and items which were chosen by less than 50% of the participants and the mean value was less than 2.5, were removed from the final model as they might be unimportant from the experts’ perspectives [39, 40]. The same procedure was performed from the first to the third round of the Delphi study.

## Results

In the first, second, and third rounds of the Delphi study, 60, 44, and 28 experts participated, respectively. The participants’ characteristics in different rounds of the Delphi study are presented in Table 1.

**Table 1:** Participants’ characteristics in the Delphi study

Variables		1st round		2nd round		3rd round	
		Frequency	Percent	Frequency	Percent	Frequency	Percent
Sex	Female	28	46.7	20	45.5	11	39.3
	Male	32	53.3	24	54.5	17	60.7
Age	≤ 30	13	21.6	12	27.3	9	32.1
	31–40	22	36.7	16	36.4	9	32.1
	> 40	25	41.7	16	36.4	10	35.8
Education	Emergency Medicine Specialist	24	40.0	14	31.9	6	21.4
	General Practitioner	12	20.0	6	13.6	6	21.4
	Ph.D.	2	3.3	2	4.5	4	14.3
	M.Sc.	5	8.3	5	11.4	5	17.9
	B.Sc.	17	28.4	17	38.6	7	25
Work experience (Years)	≤ 5	15	25.0	12	27.3	6	21.4
	6–10	25	41.7	16	36.4	8	28.6
	11–15	11	18.3	9	20.4	7	25
	> 15	9	15.0	7	15.9	7	25

As Table 1 shows, most of the participants were male in different rounds of the Delphi study, and the highest frequency was related to the participants who were over 40 years old. Most of the participants were emergency medicine specialists, and the work experience of 6 to 10 years had the highest frequency.

### 1st round of the Delphi study

The findings revealed that most of the personal data were regarded as mandatory data elements and items such as place of birth (*n* = 40, 66.7%), national identification number (*n* = 34, 56.7%), nationality (*n* = 32, 53.3%), occupation (*n* = 40, 75%), level of education (*n* = 37, 61.7%), marital status (*n* = 46, 76.7%), and data related to the patient companion and referrer (*n* = 36, 60%) were considered optional data elements. Among the personal data elements, both patient’s name and surname (4.67 ± 0.71) had the highest mean values, and the lowest mean value belonged to ethnicity (2.93 ± 1.04). In this round, the data elements that achieved a mean value between 2.5 and 3.75, and less than 75% of the participants agreed with their importance (e.g., patient date of birth, time of admission, time of discharge, contact number, and address) were entered into the 2nd round of the Delphi study. In fact, out of 27 items related to the personal data elements, 12 items were found important and 15 items were entered into the 2nd round of the Delphi study.

According to the results, most of the clinical data elements were found important and mandatory data elements. However, data items such as post-discharge recommendations (*n* = 34, 56.7%) and revised trauma score (RTS) (*n* = 31, 51.7%) were among optional data elements. Generally, among the clinical data elements, surgery (4.77 ± 0.43) had the highest mean value and financial data had the lowest mean value (3.10 ± 1.24).

Among the injury data, the highest frequency (*n* = 53, 88.3%) belonged to the body region and the lowest frequency (*n* = 37, 61.7%) was related to the time of injury. Similar to the previous section, items such as the time of injury, follow-up plan, MRI results, and ICD-based Injury Severity Score (ICISS) were entered into the 2nd round of the Delphi study, as less than 75% of the participants agreed upon the importance of these items or their mean value was between 2.5 and 3.75. At the end of the first round, out of 37 clinical data elements, 19 items were found important and included in the final model, and 18 items were entered into the 2nd round of the Delphi study.

As Table 2 presents, the data sources were divided into the hospital and non-hospital ones. Among non-hospital data sources, the highest mean value was related to the pre-hospital emergency care records (4.32 ± 0.85) and the lowest mean value was related to the municipality data (2.70 ± 1.06). As some data sources such as Forensic Medicine, Police, Ministry of Labor, Red Crescent, Ministry of Interior, Municipality, Fire Department, and the National Organization for Civil Registration did not reach a consensus, they were entered into the 2nd round of the Delphi study. In this section, out of 11 items, 3 data sources were approved and 8 data sources were entered into the 2nd round.

**Table 2:** Participants’ responses regarding the importance of data sources in the ISIS- 1st round of the Delphi study

NO	Data sources		Very important Fr (%)	Important Fr (%)	Moderately important Fr (%)	Slightly important Fr (%)	Not important Fr (%)	Mean ± SD	Median (Q1-Q3)	Consensus
65	Hospital	Emergency department records	43 (71.7)	13 (21.7)	4 (6.7)	0	0	4.65 ± 0.61	5 (4–5)	✓
66		Other Medical records (In-patient records)	43 (71.7)	13 (21.7)	4 (6.7)	0	0	4.65 ± 0.61	5 (4–5)	✓
67	Non-hospital	Pre-hospital emergency records	32 (53.3)	17 (28.3)	9 (15.0)	2 (3.3)	0	4.32 ± 0.85	5 (4–5)	✓
68		Forensic Medicine	18 (30.0)	21 (35.0)	15 (25.0)	5 (8.3)	1 (1.7)	3.83 ± 1.01	4 (3–5)	×
69		Department of Labor	5 (8.3)	11 (18.3)	28 (46.7)	12 (20.0)	4 (6.7)	3.02 ± 1.00	3 (2–4)	×
70		Red Crescent	6 (10.0)	8 (13.3)	36 (60.0)	8 (13.3)	2 (3.3)	3.13 ± 0.89	3 (3–3)	×
71		Ministry of Interior	4 (6.7)	6 (10.0)	28 (46.7)	16 (26.7)	6 (10.0)	2.77 ± 1.00	3 (2–3)	×
72		Police	10 (16.7)	20 (33.3)	22 (36.7)	8 (13.3)	0	3.53 ± 0.93	3.5 (3–4)	×
73		Municipality	4 (6.7)	6 (10.0)	27 (45.0)	14 (23.3)	9 (15.0)	2.70 ± 1.06	3 (2–3)	×
74		Fire Department	4 (6.7)	6 (10.0)	30 (50.0)	19 (31.7)	1 (1.7)	2.88 ± 0.87	3 (2–3)	×
75		National Organization for Civil Registration	7 (11.7)	8 (13.3)	29 (48.3)	16 (26.7)	0	3.10 ± 0.93	3 (2–3)	×

✓A consensus was reached

×A consensus was not reached

Regarding the functions of the ISIS, most of the suggested functions were found important by the experts (Table 3). Among them, system maintenance and updating had the highest mean value (4.52 ± 0.79), and the lowest mean value belonged to generating various statistical charts and graphs (3.72 ± 1.19). Moreover, eight functions including connection to other databases such as Police, Fire Department, Red Crescent (3.87 ± 0.89), sharing data with other organizations (3.77 ± 0.88), using clinical decision support systems (CDSS) (3.92 ± 0.96), making data available to multiple users simultaneously (3.87 ± 1.02), using geographic information system (GIS) (3.88 ± 1.12), using global positioning system (GPS) (3.82 ± 1.10), generating various statistical charts and graphs (3.72 ± 1.19) and using digital signature (3.93 ± 1.15) did not reach a consensus were entered into the 2nd round of the Delphi study. In fact, out of 30 system functions, 22 were approved and 8 were entered into the 2nd round of the study.

**Table 3:** Participants’ responses regarding the importance of the functions of the ISIS − 1st round of the Delphi study

NO	Functions of the ISIS	Very important Fr (%)	Important Fr (%)	Moderately important Fr (%)	Slightly important Fr (%)	Not important Fr (%)	Mean + SD	Median (Q1-Q3)	Consensus
76	Automated data encoding	30 (50.0)	23 (38.3)	7 (11.7)	0	0	4.38 + 0.69	4.5 (4-5)	✓
77	Quality control during data collection	29 (48.3)	23 (38.3)	6 (10.0)	2 (3.3)	0	4.32 + 0.79	4 (4-5)	✓
78	Quality control during data integration	29 (48.3)	22 (36.7)	7 (11.7)	2 (3.3)	0	4.30 + 0.81	4 (4-5)	✓
79	Searching required data	34 (56.7)	23 (38.3)	1 (1.7)	2 (3.3)	0	4.48 + 0.70	5 (4-5)	✓
80	Backing up data	33 (55.0)	23 (38.3)	2 (3.3)	2 (3.3)	0	4.45 + 0.72	5 (4-5)	✓
81	Free text data entry	27 (45.0)	21 (35.0)	5 (8.3)	4 (6.7)	3 (5.0)	4.08 + 1.12	4 (4-5)	✓
82	Connection to the trauma registry	30 (50.0)	24 (40.0)	4 (6.7)	2 (3.3)	0	4.37 + 0.76	4.5 (4-5)	✓
83	Connection to other databases such as Police, Fire Department, Red Crescent	16 (26.7)	24 (40.0)	16 (26.7)	4 (6.7)	0	3.87 + 0.89	4 (3-5)	×
84	Sharing data with other organizations	16 (26.7)	25 (41.7)	15 (25.0)	4 (6.7)	0	3.77 + 0.88	4 (3-5)	×
85	Using Clinical Decision Support Systems	20 (33.3)	20 (33.3)	15 (25.0)	5 (8.3)	0	3.92 + 0.96	4 (3-5)	×
86	Making data available to multiple users simultaneously	19 (31.7)	22 (36.7)	11 (18.3)	8 (13.3)	0	3.87 + 1.02	4 (3-5)	×
87	Access to the data Dashboard	27 (45.0)	21 (35.0)	9 (15.0)	3 (5.0)	0	4.20 + 0.88	4 (4-5)	✓
88	Hazard tracking and alerting	31 (51.7)	20 (33.3)	6 (10.0)	3 (5.0)	0	4.32 + 0.85	5 (4-5)	✓
89	Data exchange based on the standards	31 (51.7)	17 (28.3)	6 (10.0)	6 (10.0)	0	4.22 + 0.99	5 (4-5)	✓
90	Tracing injury referrals	30 (50.0)	18 (30.0)	8 (13.3)	4 (6.7)	0	4.23 + 093	4.5 (4-5)	✓
91	Using Geographic Information System	24 (40.0)	15 (25.0)	11 (18.3)	10 (16.7)	0	3.88 + 1.12	4 (3-5)	×
92	Using Global Positioning System	23 (38.3)	11 (18.3)	18 (30.0)	8 (13.3)	0	3.82 + 1.10	4 (3-5)	×
93	Generating various statistical charts and graphs	23 (38.3)	10 (16.7)	14 (23.3)	13 (21.7)	0	3.72 + 1.19	4 (3-5)	×
94	Defining new formats for reports	20 (33.3)	25 (41.7)	10 (16.7)	5 (8.3)	0	4.00 + 0.92	4 (4-5)	✓
95	Reporting	26 (43.3)	20 (33.3)	6 (10.0)	8 (13.3)	0	4.07 + 1.04	4 (4-5)	✓
96	Storing data in a regional database	27 (45.0)	23 (38.3)	10 (16.7)	0	0	4.28 + 0.74	4 (4-5)	✓
97	Storing data in the national database	30 (50.0)	19 (31.7)	11 (18.3)	0	0	4.23 + 0.77	4.5 (4-5)	✓
98	Synchronous data analysis	35 (58.3)	18 (30.0)	5 (8.3)	2 (3.3)	0	4.43 + 0.79	5 (4-5)	✓
99	Medical trends analysis	33 (55.0)	18 (30.0)	7 (11.7)	2 (3.3)	0	4.37 + 0.82	5 (4-5)	✓
100	Analyzing injury consequences	35 (58.3)	18 (30.0)	5 (8.3)	2 (3.3)	0	4.43 + 0.79	5 (4-5)	✓
101	Analyzing injury mortality	32 (53.3)	22 (36.7)	3 (5.0)	3 (5.0)	0	4.38 + 0.80	5 (4-5)	✓
102	Analyzing the degree of disability caused by injuries	32 (53.3)	20 (33.3)	7 (11.7)	1 (1.7)	0	4.38 + 0.76	5 (4-5)	✓
103	Providing secure access to online data	32 (53.3)	16 (26.7)	8 (13.3)	4 (6.7)	0	4.27 + 0.94	5 (4-5)	✓
104	Using a digital signature	27 (45.0)	12 (20.0)	11 (18.3)	10 (16.7)	0	3.93 + 1.15	4 (3-5)	×
105	System maintenance and updates	40 (66.7)	13 (21.7)	5 (8.3)	2 (3.3)	0	4.52 + 0.79	5 (4-5)	✓

✓A consensus was reached

×A consensus was not reached

### 2nd round of the Delphi study

In the 2nd round of the Delphi study, the questionnaire included 50 items that did not reach a consensus by the experts in the previous round. In this round, most of the items were found important by the respondents and some items such as citizenship (4.02 ± 0.98), occupation (4.16 ± 0.86), date of birth (3.75 ± 1.35), level of education (3.59 ± 1.33), referrer (3.50 ± 1.11), patient’s contact number (4.07 ± 1.07), patient’s address (3.73 ± 1.18), data related to the patient companion (4.07 ± 0.92), and financial data (3.98 ± 1.00) were entered into the 3rd round of the Delphi study, as they did not reach a consensus.

Among data sources, Ministry of Interior (3.75 ± 0.89), Municipality (3.68 ± 0.88), Fire Department (3.89 ± 0.93), and National Organization for Civil Registration (3.75 ± 0.99) did not reach a consensus and were entered into the 3rd round of the study. Among the proposed functions, the highest mean value (4.82 ± 0.39) belonged to using GPS and the lowest mean value (4.23 ± 0.75) belonged to using a digital signature. The participants agreed that all functions presented in the 2nd round of the Delphi study were important and could be considered in the final model.

### 3rd round of the Delphi study

In the 3rd round of the Delphi study, 13 items that did not reach a consensus in the 2nd round were questioned again (Table 4).

**Table 4:** Participants’ responses in the 3rd round of the Delphi study

NO	Data elements and data sources in ISIS		Mandatory	Optional	Very important Fr (%)	Important Fr (%)	Moderately important Fr (%)	Slightly important Fr (%)	Not important Fr (%)	Mean ± SD	Median (Q1-Q3)	Consensus
1	Personal data	Nationality	19 (67.9)	9 (32.1)	7 (25.0)	16 (57.1)	4 (14.3)	1 (3.6)	0	4.04 ± 0.74	(5 − 4) 4	✓
2		Occupation	19 (67.9)	9 (32.1)	13 (46.4)	10 (35.7)	3 (10.7)	2 (7.1)	0	4.21 ± 0.92	(5 − 4) 4	✓
3		Date of birth	20 (71.4)	8 (28.6)	16 (57.1)	6 (21.4)	3 (10.7)	2 (7.1)	1 (3.6)	4.21 ± 1.13	(5 − 4) 5	✓
4		Level of education	18 (64.3)	10 (35.7)	13 (46.4)	5 (17.9)	8 (28.6)	2 (7.1)	0	4.04 ± 1.04	(5 − 3) 4	×
5		Referrer	21 (75.0)	7 (25.0)	16 (57.1)	6 (21.4)	5 (17.9)	1 (3.6)	0	4.32 ± 0.91	(5 − 4) 5	✓
6		Contact number	22 (78.6)	6 (21.4)	18 (64.3)	7 (25.0)	3 (10.7)	0	0	4.54 ± 0.69	(5 − 4) 5	✓
7		Address	16 (57.1)	12 (42.9)	6 (21.4)	11 (39.3)	2 (7.1)	8 (28.6)	1 (3.6)	3.46 ± 1.23	(4 − 2) 4	×
8		Patient companion	15 (53.6)	13 (46.4)	8 (28.6)	9 (32.1)	0	4 (14.3)	7 (25.0)	3.25 ± 1.24	(5 − 2) 4	×
9		Financial data	19 (67.9)	9 (32.1)	14 (50.0)	8 (28.6)	2 (7.1)	2 (7.1)	2 (7.1)	4.07 ± 1.52	(5 − 4) 4	✓
10	Data sources	Ministry of Interior	-	-	13 (46.4)	3 (10.7)	5 (17.9)	5 (17.9)	2 (7.1)	3.71 ± 1.41	(5 − 2) 4	×
11		Municipality	-	-	5 (17.9)	14 (50.0)	4 (14.3)	5 (17.9)	0	3.68 ± 0.98	(4 − 3) 4	×
12		Fire Department	-	-	15 (53.6)	9 (32.1)	1 (3.6)	3 (10.7)	0	4.29 ± 0.98	(5 − 4) 5	✓
13		National Organization for Civil Registration	-	-	8 (28.6)	7 (25.0)	5 (17.9)	5 (17.9)	3 (10.7)	3.43 ± 1.37	(5 − 2) 4	×

✓A consensus was reached

×A consensus was not reached

As shown in Table 4, all data elements except the level of education, patient’s address, and the data related to the patient companion were found important and these three were removed from the final model. Among the non-hospital data sources, only Fire Department data were approved and the other three data sources, namely, the Ministry of Interior, the Municipality, and the National Organization for Civil Registration were removed from the final model.

## Discussion

In the present study, a conceptual model was developed for an ED-based ISIS to facilitate designing the system based on the users’ requirements. The findings of the present study indicated that most of the personal and clinical data elements and system functions proposed for the conceptual model were considered important by the participants. The data sources were divided into the hospital and non-hospital ones and among the non-hospital sources of data, Forensic Medicine, Police, Red Crescent, and Fire Department were found important for information sharing.

According to the literature, the availability of sufficient data in ISIS can provide a stronger context for planning and preventing injuries [13]. One of the most important and cost-effective settings for implementing an ISIS is the ED [6]. Particularly, in most low and middle income countries, emergency care and injury surveillance have been highlighted as areas which need further attentions and it is necessary to address various opportunities and challenges in these areas [14]. An ED-based ISIS helps to collect data at the very beginning of patient admission and includes all details of discharge status, recommendations for follow-up, referral to other healthcare settings, and rehabilitation centers [41]. Similarly, Lakshmi et al. regarded EDs as an opportunity to use the ISIS, since the population referred to the emergency departments is large and a number of them are injured [18]. Quigg et al. believed that ED-based ISIS is a critical part of an effective injury prevention plan, and data collection in the EDs can help to estimate burden of injuries in a given area, their nature and those groups most at risk [42]. However, as this system only focuses on the injured people, it will potentially miss injury related mortality before visiting the EDs and those injured people in the community that do not come to the hospital [14].

Compared to different types of injury surveillance systems like sports injury surveillance system [16], occupational injury surveillance system [43], and road traffic injury surveillance system [38] which have been developed for specific purposes, an ED-based ISIS can use the potentials of the EDs in terms of collecting timely and detailed data for the injured patients and store them in a central database to avoid parallel system development. This approach can also overcome data unavailability at the point of need which might be due to the distribution of data across several different systems [14]. Although injury data collection in the ED would lead to a more careful planning and decision making which, in turn, would reduce the incidence of injury, health care providers usually highlight practical issues when using information systems in the EDs [24, 25, 38]. Therefore, before designing an information system it is essential to investigate users’ requirements.

The results showed that while most of the personal and clinical data elements were considered mandatory by the participants, some items such as place of birth, national code, citizenship, occupation, level of education, and marital status were considered optional. Similarly, among clinical data elements, items such as post-discharge recommendations and adjusted trauma criteria were included in the optional data category. According to the literature, data elements can be classified differently in various settings and for different types of ISIS [27]. For example, Dinh et al. categorized data into the administrative and clinical ones [44], and Ramroop et al. categorized data elements into injury, clinical and personal/administrative data [45]. Santijiarakul et al. introduced an injury surveillance system in the context of epidemiological research which could support surveillance, investigation, and epidemiological studies [12], and Wainiqolo et al. divided all injury-related data into the demographic, injury occurrence, and hospital data [41].

In some studies, injury surveillance data have also been divided into the core and supplementary data, and a minimum data set for mandatory and optional data has been proposed under each category [10, 27]. For example, Duan et al. divided the main data elements into two categories of mandatory and optional minimum data sets [46] which are consistent with the findings of the current study.

In the present study, data sources were divided into hospital and non-hospital ones. Similarly, Holder et al. referred to hospital and non-hospital data sources which should be considered in the ISIS based on the World Health Organization’s guidelines [10]. However, as there are several data sources such as the Police and Fire Department which might include injury data, it is necessary to correctly identify which types of data and data sources need to be integrated with an ED-based ISIS [13].

As mentioned earlier, most of the functions suggested in this study were found important by the participants and included in the final model. The importance of these functions in the ISIS has also been highlighted in other studies. For example, Martinez et al. highlighted the role of data visualzation in an ISIS. This function can help to extract the required reports using advanced techniques such as magnification and filtering [47]. Providing useful reports based on the injury data is also an important function of the ISIS, which depends on data sources and data availability. In this regard, dashboards are among the useful tools that can help to provide timely and understandable reports while reducing costs and preventing data loss [48]. Other functions such as collecting, tracking, integrating, and sharing injury data, and providing reports for different levels of the healthcare system are other functions of an ISIS that have been highlighted in various studies [16, 47, 48]. Other studies have also emphasized continuous monitoring of injury data quality [8], data linkage as a basic component for improving data quality and multi-level data reporting [13, 47–50], using GIS and integration of geocodes in an ED-based ISIS [27], temporal and spatial data integration using GIS and GPS [48], and proper use of security standards [13, 16].

Overall, the results of the current study helped to develop a conceptual model for an ED-based ISIS. As Al-Hajj et al. noted, system analysts can use visual models to work collaboratively with injury stakeholders and effectively design the required systems [49]. Similarly, in the China’s national model of injury surveillance information system, Liu et al. highlighted the need for identifying data elements required for injury surveillance and monitoring its consequences. They believed that such a model can be regarded as a tool to improve quality of processes and support evidence-based healthcare policy making [11]. Therefore, it can be said that developing a conceptual model for an ED-based ISIS is an important step before designing the actual system in the future.

### Research Limitations

Although in the present study, data elements, data sources, and functional requirements of an ED-based ISIS were identified based on the experts’ perspectives, there were some limitations in the study. First of all, the number of the participants in each round of the Delphi study was limited which might be related to the coincidence of data collection with the Covid-19 pandemic. Thus, the work load of the ED staff prevented their active participation in the research. Secondly, the aim of this study was to develop a conceptual model rather than logical and physical models of the system. These models can be designed in the future research to provide a more complete documentation for a real system design. The third limitation might be related to the level of details considered for each data element. Although a large number of data elements were found important to be included in the ISIS, it was not possible to include all of them in the questionnaire. Therefore, the main data elements and functions were considered and other data elements, which have not been mentioned in this study, can be considered in the future research. Moreover, as the results showed, there were other organizations and data sources like Police, Red Crescent, and Fire Department which may benefit from data sharing. Although we were not able to include these people in the current study mainly due to the time and resource constraints, their opinions about designing and implementing an ED-based ISIS are worth investigation in the future research.

The fourth limitation might be related to the research settings which all located in the capital. In case of reaching other hospitals from other regions, the results could be different. Therefore, the results can be examined in other settings to find any possible similarities and differences.

## Conclusion

The purpose of this study was to develop a conceptual model for an ED-based ISIS. The results revealed that there was a wide range of data elements required for designing such a system and the data collected in other data sources should also be integrated into this system. This system should support analyzing data related to the injury consequences, hazard tracking and alerting, exchanging data, and tracing injury referrals. The conceptual model was presented in the current study can facilitate the process of system design in the future. In fact, the potentials of an ED-based ISIS in improving quality of care, documentation, resource allocation, and public health interventions are motivating factors to invest in this area to support injury prevention and safety. However, the final system needs to be implemented and used in practice to determine how it can meet users’ requirements.

## Supplementary Information

**Additional file 1: Appendix I.** Questionnaire

## Data Availability

The datasets used and/or analyzed during the current study available from the corresponding author on reasonable request.

## Abbreviations

ISIS: Injury Surveillance Information System
ED-based ISIS: Emergency Department- based Injury Surveillance Information System
WHO: World Health Organization
MOHP: Ministry of Health and Population
NEISS-AIP: National Electronic Injury Surveillance System-All Injury Program
CHIRPP: The Canadian Hospitals Injury Reporting and Prevention Program
