# Supplementary material for: Emergency department-based injury surveillance information system: a conceptual model
Source: BMC Emerg Med. 2023 Jun 1;23:61. doi: 10.1186/s12873-023-00831-9 (PMC10232345; doi:10.1186/s12873-023-00831-9)
Supplement: Supplementary file 1 — Additional file 1: Appendix I. Questionnaire [file 12873_2023_831_MOESM1_ESM.docx]

**Appendix I- Questionnaire**

1. Participant’s characteristics

| 1. Age: | 5. Educational level: |
| --- | --- |
| 1. Sex: Male 🞎 Female 🞎 | 6. Position: |
| 1. Field of study: | 7. Work experience: |
| 1. Work place: | 8. Email: |

1. Personal and clinical data elements of an ISIS

| No | Data element | | | Mandatory | Optional | Very important | Important | Moderately important | Slightly important | Not important |
| --- | --- | --- | --- | --- | --- | --- | --- | --- | --- | --- |
| 1 | Personal data | | Name |  |  |  |  |  |  |  |
| 2 |  |  | Surname |  |  |  |  |  |  |  |
| 3 |  |  | Father’s name |  |  |  |  |  |  |  |
| 4 |  |  | Name of the hospital |  |  |  |  |  |  |  |
| 5 |  |  | Unique patient identifier |  |  |  |  |  |  |  |
| 6 |  |  | Age |  |  |  |  |  |  |  |
| 7 |  |  | Sex |  |  |  |  |  |  |  |
| 8 |  |  | National ID |  |  |  |  |  |  |  |
| 9 |  |  | Ethnicity |  |  |  |  |  |  |  |
| 10 |  |  | Nationality |  |  |  |  |  |  |  |
| 11 |  |  | Occupation |  |  |  |  |  |  |  |
| 12 |  |  | Date of Birth |  |  |  |  |  |  |  |
| 13 |  |  | Place of Birth |  |  |  |  |  |  |  |
| 14 |  |  | Educational level |  |  |  |  |  |  |  |
| 15 |  |  | Marital status |  |  |  |  |  |  |  |
| 16 |  |  | Physician’s name |  |  |  |  |  |  |  |
| 17 |  |  | Date of admission |  |  |  |  |  |  |  |
| 18 |  |  | Time of admission |  |  |  |  |  |  |  |
| 19 |  |  | Mode of arrival to the ED |  |  |  |  |  |  |  |
| 20 |  |  | Time of arrival to the ED |  |  |  |  |  |  |  |
| 21 |  |  | Referrer |  |  |  |  |  |  |  |
| 22 |  |  | Date of discharge |  |  |  |  |  |  |  |
| 23 |  |  | Time of discharge |  |  |  |  |  |  |  |
| 24 |  |  | Type of Insurance |  |  |  |  |  |  |  |
| 25 |  |  | Contact number |  |  |  |  |  |  |  |
| 26 |  |  | Address |  |  |  |  |  |  |  |
| 27 |  |  | Patient’s companion |  |  |  |  |  |  |  |
| 28 | Clinical data | Injury data | Intent of injury |  |  |  |  |  |  |  |
| 29 |  |  | Nature of injury |  |  |  |  |  |  |  |
| 30 |  |  | External cause of injury |  |  |  |  |  |  |  |
| 31 |  |  | Activity when injured |  |  |  |  |  |  |  |
| 32 |  |  | Injury severity |  |  |  |  |  |  |  |
| 33 |  |  | Body region |  |  |  |  |  |  |  |
| 34 |  |  | Place of injury |  |  |  |  |  |  |  |
| 35 |  |  | Time of injury |  |  |  |  |  |  |  |
| 36 |  |  | Date of injury |  |  |  |  |  |  |  |
| 37 |  |  | Use of safety devices |  |  |  |  |  |  |  |
| 38 | Clinical Data | Other clinical data | Medical history |  |  |  |  |  |  |  |
| 39 |  |  | Triage data |  |  |  |  |  |  |  |
| 40 |  |  | Chief complaint |  |  |  |  |  |  |  |
| 41 |  |  | Primary diagnosis |  |  |  |  |  |  |  |
| 42 |  |  | Final diagnosis |  |  |  |  |  |  |  |
| 43 |  |  | Risk factors |  |  |  |  |  |  |  |
| 44 |  |  | Follow up plan |  |  |  |  |  |  |  |
| 45 |  |  | Financial data |  |  |  |  |  |  |  |
| 46 |  |  | Post-discharge recommendations |  |  |  |  |  |  |  |
| 47 | Clinical Data | Results and procedures | Laboratory |  |  |  |  |  |  |  |
| 48 |  |  | Ultrasonography |  |  |  |  |  |  |  |
| 49 |  |  | CT scan |  |  |  |  |  |  |  |
| 50 |  |  | MRI |  |  |  |  |  |  |  |
| 51 |  |  | CPR |  |  |  |  |  |  |  |
| 52 |  |  | Suture |  |  |  |  |  |  |  |
| 53 |  |  | Dressing |  |  |  |  |  |  |  |
| 54 |  |  | Incision |  |  |  |  |  |  |  |
| 55 |  |  | Removal |  |  |  |  |  |  |  |
| 56 |  |  | Surgery |  |  |  |  |  |  |  |
| 57 |  | Classification of injury severity | ICD-10/ICD-11 Codes |  |  |  |  |  |  |  |
| 58 |  |  | ICECI codes |  |  |  |  |  |  |  |
| 59 |  |  | Trauma and Injury Severity Score )TRISS( |  |  |  |  |  |  |  |
| 60 |  |  | Injury Severity Score (ISS) |  |  |  |  |  |  |  |
| 61 |  |  | Glasgow Coma Scale (GCS) |  |  |  |  |  |  |  |
| 62 |  |  | Abbreviated Injury Scale (AIS) |  |  |  |  |  |  |  |
| 63 |  |  | Revised Trauma Score (RTS) |  |  |  |  |  |  |  |
| 64 |  |  | ICD-based Injury Severity Score (ICISS) |  |  |  |  |  |  |  |

C. Data sources

| NO | Data sources | | Very important | Important | Moderately important | Slightly important | Not important |
| --- | --- | --- | --- | --- | --- | --- | --- |
| 65 | Hospital | Emergency department records |  |  |  |  |  |
| 66 |  | Other Medical records (In-patient records) |  |  |  |  |  |
| 67 | Non-hospital | Pre-hospital emergency records |  |  |  |  |  |
| 68 |  | Forensic Medicine |  |  |  |  |  |
| 69 |  | Department of Labor |  |  |  |  |  |
| 70 |  | Red Crescent |  |  |  |  |  |
| 71 |  | Ministry of Interior |  |  |  |  |  |
| 72 |  | Police |  |  |  |  |  |
| 73 |  | Municipality |  |  |  |  |  |
| 74 |  | Fire Department |  |  |  |  |  |
| 75 |  | National Organization for Civil Registration |  |  |  |  |  |

1. System functions

| NO | Functions of an ISIS | Very important | Important | Moderately important | Slightly important | Not important |
| --- | --- | --- | --- | --- | --- | --- |
| 76 | Automated data encoding |  |  |  |  |  |
| 77 | Quality control during data collection |  |  |  |  |  |
| 78 | Quality control during data integration |  |  |  |  |  |
| 79 | Searching required data |  |  |  |  |  |
| 80 | Backing up data |  |  |  |  |  |
| 81 | Free text data entry |  |  |  |  |  |
| 82 | Connection to the trauma registry |  |  |  |  |  |
| 83 | Connection to other databases such as Police, Fire Department, Red Crescent |  |  |  |  |  |
| 84 | Sharing data with other organizations |  |  |  |  |  |
| 85 | Using clinical decision support systems |  |  |  |  |  |
| 86 | Making data available to multiple users simultaneously |  |  |  |  |  |
| 87 | Access to the data Dashboard |  |  |  |  |  |
| 88 | Hazard tracking and alerting |  |  |  |  |  |
| 89 | Data exchange based on the standards |  |  |  |  |  |
| 90 | Tracing injury referrals |  |  |  |  |  |
| 91 | Using geographic information system |  |  |  |  |  |
| 92 | Using global positioning system |  |  |  |  |  |
| 93 | Generating various statistical charts and graphs |  |  |  |  |  |
| 94 | Defining new formats for reports |  |  |  |  |  |
| 95 | Reporting |  |  |  |  |  |
| 96 | Storing data in a regional database |  |  |  |  |  |
| 97 | Storing data in the national database |  |  |  |  |  |
| 98 | Synchronous data analysis |  |  |  |  |  |
| 99 | Medical trends analysis |  |  |  |  |  |
| 100 | Analyzing injury consequences |  |  |  |  |  |
| 101 | Analyzing injury mortality |  |  |  |  |  |
| 102 | Analyzing the degree of disability caused by injuries |  |  |  |  |  |
| 103 | Providing secure access to online data |  |  |  |  |  |
| 104 | Using a digital signature |  |  |  |  |  |
| 105 | System maintenance and updates |  |  |  |  |  |
